# Effects of Acute Red Spinach Powder (VitaSpinach^®^) Ingestion on Muscular Endurance and Resistance Exercise Performance

**DOI:** 10.3390/muscles4040060

**Published:** 2025-12-03

**Authors:** Haley M. Nguyen, Sophia L. Porrill, Rebecca R. Rogers, Josselyn Jose-Gomez, Rachel E. Wright, Phoebe N. Spears, Christopher G. Ballmann

**Affiliations:** 1 Department of Human Studies, University of Alabama at Birmingham, Birmingham, AL 35294, USA; nguyenhm@uab.edu (H.M.N.); sporrill@uab.edu (S.L.P.); jjosegom@uab.edu (J.J.-G.); wrighte@uab.edu (R.E.W.); pnspears@uab.edu (P.N.S.); 2 Department of Family and Community Medicine, University of Alabama at Birmingham, Birmingham, AL 35294, USA; rrrogers@uab.edu; 3 Department of Physical Therapy, University of Alabama at Birmingham, Birmingham, AL 35294, USA

**Keywords:** nitrate, nitrite, bench press, rating of perceived exertion, velocity

## Abstract

**Introduction:** Red spinach powder (RSP) contains high amounts of inorganic nitrate/nitrite (NO_3_/NO_2_), which has been suggested to alter vascular activity, cognitive processing, and sprint exercise performance. There have been few investigations as to whether RSP serves as an ergogenic aid to improve resistance exercise performance, particularly muscular endurance. **Purpose:** The purpose of this study was to investigate acute RSP (VitaSpinach^®^) supplementation on muscular endurance and velocity during bench press exercise. **Methods:** In a double-blind, counterbalanced crossover manner, resistance-trained males (n = 14) were subjected to two supplement conditions as follows: (1) placebo (PL; purple sweet potato) or (2) red spinach powder (RSP; 400 mg NO_3_). Supplements were consumed 2 h prior to exercise and blood was collected immediately pre-exercise to determine NO_3_/NO_2_ levels. To determine barbell velocity, participants completed two sets × two repetitions with maximal effort, while a rotary encoder measured mean barbell velocity. Following this, participants performed three sets × repetitions to exhaustion (RTE) at 60% of 1-Repetition Maximum (1-RM), separated by 2 min of rest, to determine muscular endurance. Local (lRPE) and global (gRPE) ratings of perceived exertion were measured after exercise. Blood NO_3_/NO_2_, RTE, mean velocity, lRPE, and gRPE were compared between supplement conditions. **Results:** RSP resulted in significantly higher blood levels of total NO_3_/NO_2_ (*p* < 0.001) compared to PL. RSP did not result in superior total RTE (*p* = 0.935) but increased mean velocity (*p* = 0.035) compared to PL. Both lRPE (*p* = 0.027) and gRPE (*p* = 0.028) were significantly reduced with RSP supplementation. **Conclusions:** Findings suggest acute RSP ingestion increased NO_3_/NO_2_ and bench press velocity. While muscular endurance remained unchanged, RSP resulted in lower perceptions of exertion.

## 1. Introduction

Red spinach (*Amaranthus dubius*) is a leafy vegetable that is widely cultivated in African and Asian regions [1]. Aside from culinary uses, extracts from red spinach have been anecdotally used in folk medicine with purported benefits towards immunity, gut health, and as a source of iron enrichment. Red spinach has a high mineral, flavonoid, and antioxidant content, which may suppress oxidative stress, inflammation, and provide anticarcinogenic effects [2]. It is also rich in inorganic nitrate (NO_3_) and has been repeatedly shown to result in increased NO_3_ and associated metabolites in human plasma, including nitrite (NO_2_) and nitric oxide (NO) [3,4]. Formation of NO induces a wide array of physiological changes, including relaxation of smooth muscle in vasculature [5], altered blood flow [6], enhanced oxygen kinetics [7], and metabolic efficiency [8]. These effects have caused considerable attention in the application of NO_3_ supplementation in sports and athletics, with a myriad of evidence supporting ergogenic effects of dietary NO_3_ enrichment [9,10,11]. However, as a source of dietary NO_3_, red spinach has been comparatively less studied, and its effects on resistance exercise and muscular endurance are still being elucidated.

With regard to resistance exercise, acute inorganic NO_3_ supplementation has been primarily studied in the context of beetroot juice or other non-red spinach sources [9,12,13]. Williams et al. showed that a single dose of beetroot juice (400 mg NO_3_) in resistance-trained males increased bench press repetition volume and explosive ability reflected in higher barbell velocity [9]. Furthermore, Ranchal-Sanchez et al. showed a single dose of beetroot juice (400 mg NO_3_) enhanced lower-body muscular endurance during squat exercise in healthy males [12]. Acute beetroot juice ingestion (800 mg NO_3_) has also been linked to enhanced peak and mean power development during concentric and eccentric portions of movement during lower-body resistance exercise [13]. The physiological underpinnings of reported improvements have been suggested to be related enhanced phosphocreatine resynthesis, attenuation of factors related to muscular fatigue, increased muscle shortening velocity [14]. However, whether red spinach delivers similar ergogenic effects as a dietary NO_3_ source is not fully clear.

Some reports have suggested that red spinach ingestion enhances exercise performance, although some reports are conflicting [3,15,16,17]. Gonzalez et al. showed that seven days of daily red spinach ingestion (90 mg NO_3_) resulted in improved power output and 4 km endurance cycling test times in recreationally active men and women [15]. Importantly, these improvements were concomitant to improvements in muscular fatigue, reflecting improved muscular endurance. Bolstering these findings, Moore et al. showed that a single acute dose of red spinach (90 mg NO_3_) before exercise improved ventilatory threshold during a graded maximal test, suggesting that initiation of accelerated anaerobic metabolism may be attenuated with red spinach, thus diminishing fatigue [16]. Supporting this, Raymond et al. showed that a single dose of red spinach (400 mg NO_3_) lowered power output loss and fatigue during repeated sprinting, with accompanying increases in post-exercise blood lactate, suggesting improved lactate clearance [3]. Consequently, others have shown little to no improvement in exercise performance following red spinach ingestion. For example, Townsend et al. showed that eight weeks of daily red spinach ingestion (180 mg NO_3_) did not improve body composition or Wingate sprint performance in male baseball athletes [18]. While disparities in findings are not fully clear, it is likely that differences in supplement timing, dosage, and type of accompanying exercise influence the efficacy of red spinach [19]. Thus, more studies of the optimal supplementation regimens with red spinach across different exercise modes is warranted.

To date, there is limited evidence to inform the efficacy of acute red spinach ingestion on resistance exercise performance and muscular endurance. Haynes et al. reported that seven days of red spinach ingestion (180 mg NO_3_) did not influence repetition volume or power development during upper-body resistance exercise in resistance-trained males [20]. The authors also noted no changes in muscle oxygenation or cognitive performance. However, the red spinach supplement used was given in capsule form, which likely bypassed NO_3_ conversion by oral microbiota. Since NO_3_ is largely reduced in the mouth and the elimination of this oral reduction in NO_3_ negates supplement benefits [21], ingestion via capsule form may not impart increases in NO, which is critical for ergogenic effects. Furthermore, numerous investigations showing the efficacy of acute dietary NO_3_ ingestion on exercise performance have used single doses containing 400–800 mg NO_3_, which is markedly higher than that used by Haynes et al. [3,9,12,13,18]. The aim of this study was to investigate the effects of acute red spinach powder (RSP; 400 mg NO_3_) ingestion on muscular endurance and performance during upper-body resistance exercise. We hypothesized that acute RSP would result in improved upper-body muscular endurance and resistance exercise performance.

## 2. Results

### 2.1. Plasma Nitrate/Nitrite (NO_3_/NO_2_)

**Figure 1:**
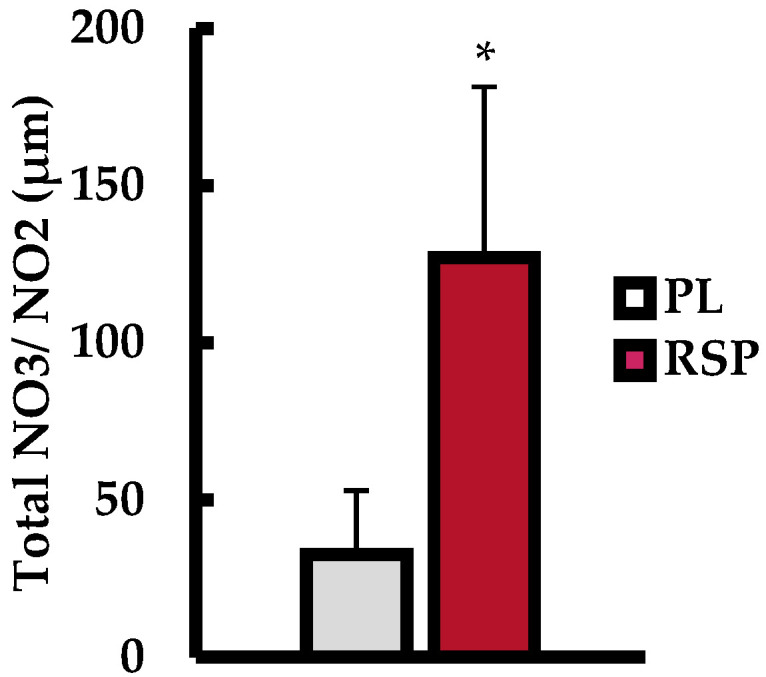
Total plasma nitrate/nitrite (NO_3_/NO_2_) between placebo (PL; gray bars) and red spinach powder (RSP; red bars) 2 h post-ingestion. Data are presented as mean ± SD. * indicates statistically different from PL (*p* ≤ 0.05).

Total plasma NO_3_/NO_2_ (μm) measurements are displayed in Figure 1. Analysis revealed that RSP ingestion resulted in significantly higher plasma NO_3_/NO_2_ concentrations compared to PL (PL = 32.6 μm ± 20.2; RSP = 127.1 μm ± 54.2; *p* < 0.001; rbb = +1.0).

### 2.2. Repetitions to Exhaustion (RTE) and Mean Velocity

**Figure 2:**
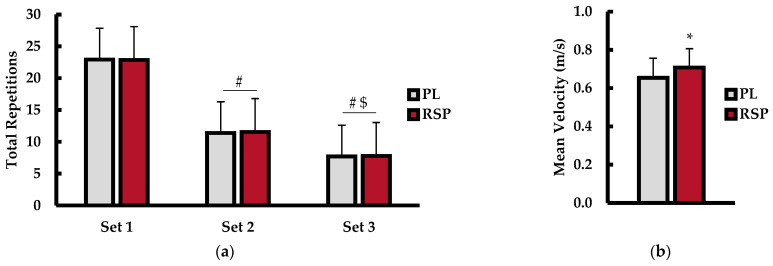
(**a**) Set-to-set and total repetitions to exhaustion; (**b**) mean barbell velocity (m·s^−1^) between placebo (PL; gray bars) and red spinach powder (RSP; red bars) treatment during explosive bench press exercise. Data are presented as mean ± SD. # indicates statistically different from Set 1 (*p* ≤ 0.05). $ indicates statistically different from Set 2 (*p* ≤ 0.05). * indicates statistically different from PL (*p* ≤ 0.05).

Repetitions to exhaustion (repetitions) are shown in Figure 2a. There was no main effect for treatment (*p* = 0.935; η^2^ < 0.001), but a main effect for set (*p* < 0.001; η^2^ = 0.837) was observed. No interaction for condition × set (*p* = 0.951; η^2^ < 0.001) was observed. Post hoc analysis for set revealed that participants completed a higher number of repetitions throughout the first set versus the second (Set 1 = 23 reps ± 4; Set 2 = 11 ± 1; *p* < 0.001; d = 3.1) and third (Set 3 = 8 reps ± 2; *p* < 0.001; d = 3.3) sets, regardless of treatment. Furthermore, greater repetitions were accomplished during the second set (*p* = 0.001; d = 1.7) versus the third set, irrespective of condition. Mean velocity (m·s^−1^) of the barbell is displayed in Figure 2b. Analysis showed that RSP treatment resulted in significantly higher mean velocity (PL = 0.66 m·s^−1^ ± 0.10; RSP = 0.71 m·s^−1^ ± 0.95; *p* = 0.035; d = 0.63) compared to PL treatment.

### 2.3. Global (gRPE) and Local (lRPE) Ratings of Perceived Exertion

**Figure 3:**
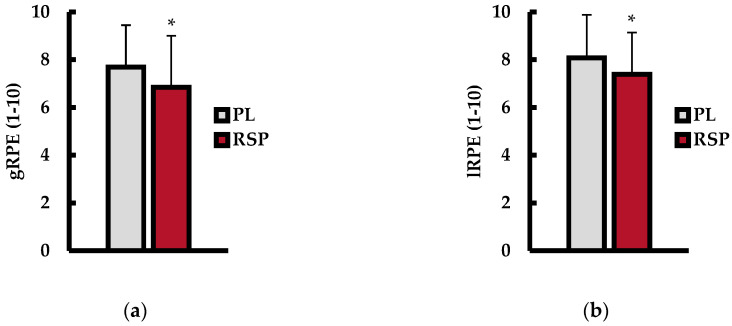
(**a**) Global rating of perceived exertion (gRPE); (**b**) local rating of perceived exertion (lRPE) between placebo (PL; gray bars) and red spinach powder (RSP; red bars) treatment following repeated bench press exercise. Data are presented as mean ± SD. * indicates significant differences from PL (*p* ≤ 0.05).

Global rating of perceived exertion (gRPE; 1–10 scale) is shown in Figure 3a. Analysis revealed that gRPE was significantly lower with RSP ingestion compared to PL (PL = 7.7 a.u. ± 1.6; RSP = 6.9 a.u. ± 2.0; *p* = 0.028; d = 0.66). Local rating of perceived exertion (lRPE; 1–10 scale) is shown in Figure 3b. RSP ingestion resulted in lower lRPE compared to PL (PL = 8.0 a.u. ± 1.7; RSP = 7.2 a.u. ± 1.7; *p* = 0.027; d = 0.65).

## 3. Discussion

Acute ingestion of dietary NO_3_ has been widely reported to impart ergogenic effects during resistance exercise [9,12,13]. While red spinach ingestion has been implicated to increase NO_3_ and associated metabolites [3,4], its effects on resistance exercise performance remain unclear. Current findings suggest that acute ingestion of RSP results in increased NO_3_/NO_2_ in resistance-trained males. Furthermore, RSP resulted in increased barbell velocity during bench press but had little effect on repetition volume and muscular endurance. However, perceptions of exertion were significantly lower with RSP ingestion. Overall, these findings suggest RSP is a viable source of dietary NO_3_, an ergogenic aid for explosive performance, and modulator of perceived exertion during resistance exercise. While mechanisms for these effects are not fully clear, RSP appears to be an effective dietary source of NO_3_ with performance-enhancing benefits.

Currently, RSP ingestion resulted in increased mean barbell velocity during explosive bench press exercise. Although this has not been previously reported with RSP as the primary source of NO_3_, similar findings utilizing other forms of dietary NO_3_ support this. Williams et al. showed that enhanced bench press velocity with acute beetroot juice (400 mg NO_3_) ingestion similarly in resistance-trained males [9]. Furthermore, Rodriguez-Fernandez et al. showed that acute beetroot juice ingestion (800 mg NO_3_) resulted in increased power output during both concentric and eccentric phases of lower-body resistance exercise [13]. Although the physiological underpinning for the effects of RSP on mean velocity is not yet fully delineated, enhancement of muscle contractile force has been previously described as a byproduct of increased NO production. Indeed, previous reports from animal studies have shown increased contractile force of muscle, particularly fast-twitch muscle types, which has been attributed to changes in muscle proteins responsible for calcium handling [22]. While increased contractile force of muscle has been confirmed in humans with NO_3_ ingestion, it appears it may be mediated by different mechanisms, as alterations in calcium-handling proteins are largely unsupported in humans [23]. Regardless, the acute nature of the current supplementation protocol is unlikely to result in protein accumulation that would meaningfully affect performance. However, other evidence suggests that increases in NO from NO_3_ supplementation may increase nitrosylation of muscle ryanodine receptors, thereby increasing calcium release and sensitivity [24]. This may result in greater contractile function through increasing twitch force, rate of force development, and maximal shortening velocity [24]. While caution is warranted since mechanisms are largely speculative at this time, increases in NO from RSP ingestion may alter calcium levels in active musculature, leading to enhanced cross-bridge formation, allowing for greater movement velocity. Future mechanistic studies will be necessary to confirm or refute this, since it was not directly measured.

Contrary to the current hypotheses, acute ingestion of RSP did not result in improvements in muscular endurance as evidenced by the lack of changes in repetition volume. The lack of ergogenic effects toward muscular endurance with red spinach are supported by previous evidence [20]. Haynes et al. similarly showed no improvements in repetition volume during bench press with seven days of red spinach ingestion (180 mg NO_3_) [20]. However, this stands in contrast to findings with other forms of dietary NO_3_ which have shown improvements in maximal repetitions [9,12]. Reasons for disparities are not fully clear at this time. Previous investigations utilizing other forms of NO_3_ have shown or speculated changes in phosphocreatine recovery and efficiency with NO_3_ ingestion due to enhanced oxygen kinetics [25]. This may, in turn, result in improved muscular endurance, especially during repeated exercise. However, Haynes et al. showed little to no effect of red spinach on muscle oxygenation during resistance exercise [20]. Thus, red spinach may not be effective at increasing oxygen kinetics in skeletal muscle and may not modulate metabolism for muscular endurance. Interestingly, local and global RPE were significantly lower with RSP ingestion despite similar repetition volume as placebo. These findings are supported by previous evidence showing lower local and global RPE during lower-body exercise following five days of daily beetroot juice (400 mg NO_3_) ingestion [26]. Furthermore, leg muscle pain during exercise was also dampened with NO_3_ ingestion. While largely speculative at this time, it is plausible that RSP ingestion modulated nociceptive signaling similarly during exercise, thereby making the participants perceive that they were exerting less. Furthermore, it is possible that increases in vasodilation in working skeletal muscle via NO [27], as a consequence of RSP ingestion, may have aided in less hydrogen ion accumulation and pain sensation during exercise [28]. However, since pH changes in muscle largely reflect fatigue during endurance tasks and no changes in muscular endurance were currently observed, this may be unlikely. Further investigation into fatigue contributors during exercise with NO_3_ supplementation is warranted. From a practical standpoint, perceptual responses such as RPE may be a limiting factor in exercise tolerance [29]. Therefore, it is plausible that lowering RPE with RSP ingestion may improve exercise tolerance even in the absence of increased muscular endurance, but further research is needed to confirm this.

While the current investigation provides novel information regarding RSP ingestion and resistance exercise performance, there were a number of limitations. The sample population tested was small and similar in characteristics. Thus, it is unknown if current findings will translate to larger populations with differing characteristics such as age, training experience, and biological sex. Furthermore, the physiological mechanisms underlying the effects of RSP remain either speculative or unclear. Future research using more reductionist study designs will be necessary to fully understand how RSP influences skeletal muscle function and metabolism. Lastly, only a single upper-body exercise and load were assessed. It is possible that exercises involving the lower body or higher loads may elicit differing responses. Future studies should integrate different exercises and loads to give a more comprehensive understanding of how RSP influences resistance exercise.

## 4. Materials and Methods

### 4.1. Study Design

This investigation used a double-blinded, randomized, crossover, counterbalanced study design. Following informed consent, each participant completed three visits, including two visits with varying treatment conditions: (1) placebo (PL; purple sweet potato powder) and (2) red spinach powder (RSP; VitaSpinach^®^ Nutrigardens; Portland, OR, USA). On the first visit, participants performed a one-repetition maximum (1-RM) test for the bench press exercise to determine maximal strength. During the subsequent two visits, participants consumed a single dose of the corresponding treatment 2 h prior to a pre-exercise blood collection (to measure NO_3_/NO_2_), followed by performing the bench press with maximum explosive intent to measure barbell velocity, and followed by three sets of repetitions to exhaustion (RTE) to assess muscular endurance. Post-exercise measurements of local (chest) and global (overall) ratings of perceived exertion (RPE) were recorded. Comparisons were drawn between treatment conditions, and all study visits were separated by a minimum 48 h washout period.

### 4.2. Participants

Using G-power 3.1.9.6 open-access software [30], adequate sample size was determined via a prior power analysis. A previous investigation showed improvements in muscular endurance (i.e., lower muscular fatigue) during endurance cycling exercise with RSP supplementation, with an effect size of f = 0.369 [15]. Therefore, sample size was determined to detect possible changes in muscular endurance during repeated bench press using the following parameters: test = repeated-measures f-test, f = 0.369, groups = 2, measurements = 3, α = 0.05, and β = 0.8. This yielded a total sample size of n = 14. Therefore, fourteen resistance-trained males volunteered to participate in the study, and their descriptive characteristics are shown in Table 1. To be considered resistance-trained, individuals had to engage in weight training > 2 days/week [31]. Participants reported no musculoskeletal injuries in the past six months that limited exercise ability and no chronic diseases. A physical activity readiness questionnaire was used to determine the safety of participation in exercise [31]. Participants were asked to refrain from anti-bacterial mouth wash, caffeine, nicotine, pre-workout supplements, and alcohol 12 h prior to each visit, and vigorous upper-body exercise for 24 h prior to each visit [31]. Participants were not currently supplemented with inorganic nitrate or equivalents at the time of participation and were asked to replicate dietary and sleep habits to the greatest extent possible prior to each visit. Verbal and written informed consent were obtained from each participant, and all experimental procedures were conducted in accordance with the Declaration of Helsinki and approved by the University of Alabama at Birmingham Institutional Review Board (IRB) (UAB IRB-300013510; 28 January 2025).

### 4.3. One-Repetition Maximum (1-RM) and Familiarization

A one-repetition maximum (1-RM) test for the bench press exercise was conducted on the first visit for each participant, as previously described by Ballmann et al. [32]. To begin, participants completed a warm-up with five repetitions at 40%, followed by three repetitions at 60% of self-reported 1-RM. Following the warm-up, the weight of the barbell was progressively increased by 2.5–20.0 kg until the participant could not complete the lift [33]. Attempts were separated by 5 min of rest. To familiarize with lifting explosively, a 20 kg Olympic barbell was lifted with maximum explosive intent [34]. This was also reinforced during the warm-up sets for subsequent visits. Exercise form was corrected and demonstrated if needed. Load percentages for subsequent visits were determined using the 1-RM load obtained.

### 4.4. Supplementation and Plasma NO_3_/NO_2_

For the RSP condition, participants consumed a single dose of 4.4 g of pure red spinach powder (VitaSpinach^®^, Nutrigardens; Portland, OR, USA), which was standardized to deliver an NO_3_ content of ~400 mg [3]. For the PL treatment, purple sweet potato powder (ORGFUN, HAWK ON ARRIVAL; North Haledon, NJ, USA) was used to mimic RSP color/form and was given in an identical manner. Treatments were distributed in opaque containers coded to blind both participants and researchers to treatment identity. Treatments were dissolved in water and ingested 2 h prior to testing as previous evidence has suggested that this timing corresponds to peak plasma NO_3_ levels [9].

Analysis of total plasma NO_3_/NO_2_ was completed as previously reported by our lab [3]. A capillary blood sample (~500 μL) was collected upon arrival, occurring 2 h after the treatment ingestion, through a finger stick [35]. A 2.0 mm depth blade lancet (17 gauge) was used to induce bleeding on the finger and whole blood was collected into potassium- EDTA-coated microvette^®^ tubes (SARSTEDT, Newton, NC, USA). Plasma was decanted from whole blood following centrifugation at 10,000 rpm for 10 min. Plasma samples were then frozen at −80 °C until the end of data collection, when analysis was performed. Total NO_3_/NO_2_ in plasma samples were detected using a commercially available biochemical detection kit (Cayman Chemical; Ann Abor, MI, USA) [3,36,37]. Each plasma sample was analyzed in duplicate and methods were followed according to the manufacturer’s directions.

### 4.5. Procedures

During the second and third visit, participants performed a series of bench press exercise tests 2 h after treatment ingestion, as previously described by Williams et al. [9]. A standardized warm-up consisting of five repetitions at 40% 1-RM followed by three repetitions at 50% 1-RM was first completed. Warm-up sets were separated by 3 min of rest. Following this, explosive ability was assessed via mean velocity by completing two sets × two repetitions at 60% 1-RM as explosively as possible, with 2 min of rest between sets. For these sets, mean velocity of the barbell was detected using a rotary encoder (GymAware; Kinetic Performance Technology, ACT, Australia) [38,39]. The highest average velocity was used for performance analysis. After another 2 min rest period, participants completed three sets × repetitions to exhaustion (RTE) at 60% 1-RM, separated by 2 min of rest to assess muscular endurance. Total and per-set repetition volumes were used for performance metrics. After exercise, lRPE (i.e., chest) and gRPE (i.e., overall) were documented using a 1–10 scale.

### 4.6. Data Analysis

Statistical analyses were conducted by the means of Jamovi software (Version 0.9) [40,41]. All data are presented as mean ± standard deviation (SD). The Shapiro–Wilk method was used to detect data normality. To determine differences in muscular endurance (i.e., repetition volume), a two × three [treatment × set] repeated-measures ANOVA was used. For significant main effects and interactions, pairwise comparisons were conducted using a Tukey post hoc analysis. For main effects and interactions, estimates of effect size are shown as eta squared (η^2^). To determine differences in mean velocity, gRPE, and lRPE, a paired-samples t-test was used. Estimates of effect size for t-tests are shown as Cohen’s d [42]. Data from plasma NO_3_/NO_2_ violated assumptions of normality, and a non-parametric t-test (Wilcoxon rank) was used to determine differences, with rank-biserial correlation (rbb) as an estimate of effect size. Significance was set at *p* ≤ 0.05.

## 5. Conclusions

In conclusion, acute ingestion of RSP (400 mg NO_3_) enhances circulating NO_3_/NO_2_ and explosive resistance exercise performance in resistance-trained males. However, RSP has limited impact on muscular endurance during repeated bench press exercise. Furthermore, ingestion of RSP may alter perceptions of exertion during exercise. From a practical standpoint, consuming RSP may have several advantages compared to other forms of dietary NO_3_, including higher NO_3_ concentration per weight of product, lower sugar content, and higher mineral enrichment. Furthermore, phytochemical and polyphenol content of RSP may be of some benefit to inflammation, albeit not the focus of the current study. Since RSP comes in a readily dissolvable powder and has a relatively stable shelf-life, RSP may be more convenient for athletes to consume compared to traditional beetroot juice, especially when traveling. While mechanisms underlying the benefits of RSP are still being elucidated, current results support previous findings in other forms of NO_3_, suggesting that RSP may impart similar physiological changes; however, further study is needed to confirm this.

## Figures and Tables

**Table 1:** Descriptive characteristics of participants.

Descriptive Characteristics (n = 14)	Mean ± SD
Age (years)	23.1 ± 6.3
Height (cm)	174.2 ± 11.7
Body Mass (kg)	76.7 ± 10.4
RT Experiences (years)	6.4 ± 6.9
1-RM (kg)	104.3 ± 20.7
Relative 1-RM (1-RM/Body mass)	1.4 ± 0.2

Note: resistance training (RT); one-repetition maximum (1-RM).

## Data Availability

The original contributions presented in this study are included in the article. Further inquiries can be directed to the corresponding author.
